# The Impact of Brain Tumors on Emotional and Behavioral Functioning

**DOI:** 10.7759/cureus.75315

**Published:** 2024-12-08

**Authors:** Rayyan R Samman, Jumana H Timraz, Ahmed Mosalem Al-Nakhli, Shyma Haidar, Qalbe Muhammad, Husna Irfan Thalib, Ahmed Hafez Mousa, Mohammad Samy Kharoub

**Affiliations:** 1 General Medicine Practice Program and Surgery, Batterjee Medical College, Jeddah, SAU; 2 Department of Neurosurgery, Rashid Hospital, Dubai Health, Dubai, ARE; 3 Department of Neurosurgery, Graduate Medical Education, Mohammed Bin Rashid University of Medicine and Health Sciences, Dubai Health, Dubai, ARE; 4 Department of General Surgery, General Medicine Practice Program and Surgery, Batterjee Medical College, Jeddah, SAU

**Keywords:** behavioural, behavioural functioning, brain tumour, emotional wellness, neurosurgery

## Abstract

While the physical manifestations of brain tumors are well-documented, their impact on the emotional and psychological landscape of patients is of equal importance. Patients frequently experience a range of challenges from depression, apathy, and increased aggression to personality changes. The complexity of these changes and their effects on emotional functioning are shaped by tumor characteristics, including location, growth rate, and the corresponding hormonal imbalances. These challenges may ripple outward, affecting not only the patients themselves but also their caregivers. This review aims to examine the diverse emotional experiences associated with various brain tumor types and locations, through understanding the neurobiological mechanisms underlying these changes. The impact of psychosocial factors on emotional distress and coping strategies is also explored, focusing on the critical role of social support and resilience. The need for integrated care that addresses both the physical and psychological aspects of brain tumors is essential for improving the quality of life (QoL) for patients and their families. The close relationship between emotional and cognitive difficulties is analyzed, stressing how these challenges can mutually reinforce each other, creating a convoluted and challenging situation for brain tumor patients. By understanding and addressing these issues, healthcare providers can better support patients and improve their overall QoL. This review seeks to consolidate the current understanding of this complicated relationship, drawing from an array of studies, reviews, and meta-analyses.

## Introduction and background

It is without question that the diagnosis of any cancer will trigger a period of emotional turmoil. The insidious nature of brain tumors specifically represents a significant challenge in oncology to both medical professionals and those affected, as the very essence of an individual’s personality and cognitive function may potentially be disrupted. Though brain tumors account for a small percentage of overall cancers, over 300,000 individuals worldwide are diagnosed annually; two-thirds of which face a grim prognosis, with many experiencing significant functional limitations [1]. Albeit the physical manifestations of these tumors are usually the primary focus of clinical attention, the emotional and psychological consequences demand comprehensive understanding. Optimal management of brain tumors necessitates an approach that not only targets the disease itself but also addresses the emotional challenges that patients may encounter throughout their illness journey. Emerging research entails the distinct disease trajectory associated with primary brain tumors, indicating that patients undergo notable unpredictable changes in their neurological functioning [2]. Consequently, a major decline in their social, family, and occupational obligations can be observed [3]. A variety of mood disorders such as depression and anxiety are a common concern for patients, with rates exceeding those observed in healthy controls or those with cancer outside the central nervous system [4]. Interestingly, the reported prevalence of these disorders is highly variable, suggesting that tumor-unique factors influence the development and severity of these disorders [5]. Studies show that health-related quality of life (HRQoL) in patients with both low-grade glioma (LGG) and high-grade glioma (HGG) are comparable, making it clear that the impact of HRQoL is not solely determined by tumor grade [6]. Rather the course of the tumor, whether it progresses or remains stable, the location of the tumor within the brain, its biological behavior, and the corresponding treatments employed play a critical role in this variability [7,8]. Some patients may struggle with negative thought patterns hindering their emotional well-being. Adapting to the challenges of a brain tumor calls for patients to actively manage their behaviors and emotions while maintaining cognitive flexibility, allowing them to adjust their perspectives and strategies as needed. Studies demonstrate that patients express resilience by adopting positive coping mechanisms, focusing on addressing the stressor head-on, and managing emotional responses [9]. Thus, diverse avenues need to be explored to strengthen resilience and empower patients as they navigate through the unique challenges of living with a brain tumor.

## Review

Relationship between brain tumors and emotional functioning: current understanding

Tumor characteristics, including growth rate and location along with their hormonal implications, can significantly impact the patient’s emotional well-being, and overall QoL. Slow-growing tumors, such as meningiomas or low-grade gliomas, allow for greater brain plasticity, resulting in more focal neurocognitive deficits, leading to specific impairments. The brain's plasticity might allow for gradual and initially subtle changes in behavior, often recognized only in the later stages [10]. Cognitive deficits in memory, attention, and executive functions are commonly observed in these individuals, especially when situated in the frontal lobe. In contrast, aggressive tumors in cases of HGG limit the brain's capacity to adapt due to their rapid growth and infiltration. This may lead to a combination of focal and diffuse neurocognitive deficits (both specific and widespread impairments). Emotional disturbances such as depression and fatigue are prevalent in these patients [11]. As these aggressive diseases advance, the burden of coping with these changes often becomes overwhelming, leading to psychosocial issues such as depression, anxiety, and social isolation. Pituitary adenomas also contribute to cognitive and emotional challenges in brain tumor patients, leading to cognitive deficits in memory and executive functions, accompanied by psychological disturbances of varying degrees depending on the hormonal-mediated effect [11].

**Figure 1:**
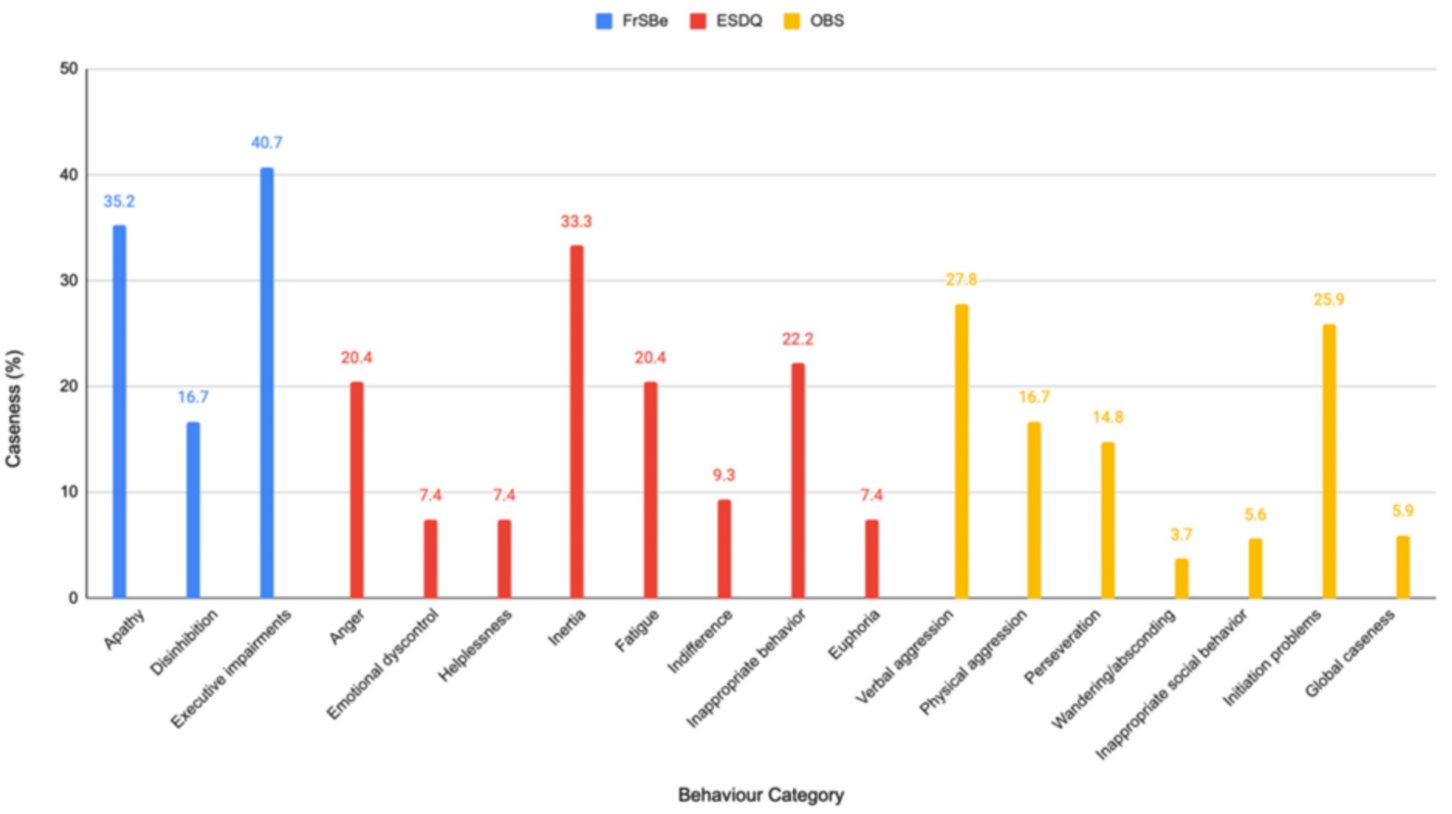
Comparison of Behavioural Assessments in Brain Tumour Patients Image credits: Qalbe Muhammad; tool used: Microsoft PowerPoint

A study by Simpson et al. [12] emphasized the prevalent behavioral changes seen across all tumor grades among 54 patients by using three different scales of measurement. The Frontal System Behaviour Scale (FrSBe) encompasses three behavioral dimensions; the first of which is apathy, manifesting as diminished motivation, initiative, and emotional responsiveness. Disinhibition, being the second domain, alludes to impulsivity along with socially inappropriate behavior, including aggression, inability, and emotional lability. Lastly, executive dysfunction is characterized by difficulties with planning, organization, problem-solving, and irritability. Meanwhile, the range of emotional and social difficulties was measured using the Emotion and Social Dysfunction Questionnaire (ESDQ) via in-depth, semi-structured interviews. To obtain a comprehensive understanding of behavioral shifts observed in patients’ daily lives, both patients and their designated family members/caregivers were asked to complete the FrSBe and ESDQ, facilitating a direct comparison between the patient's self-perception and the observations made by their close caregivers. Finally, to enhance the comprehensiveness of the assessment, healthcare professionals directly involved in the patient’s care contributed their independent evaluations of observed behavioral changes using the Overt Behaviour Scale (OBS). Although these symptoms may be experienced by all patients to some extent, Figure 1 shows the presence of clinically significant levels of symptoms on each scale. The percentage of patients meeting this criterion is referred to as “caseness” [12].

Executive impairments, apathy, and disinhibition were relatively common in this patient group with 40.7%, 35.2%, and 16.7% meeting the criteria for caseness respectively. Inertia, indicating a lack of motivation, and fatigue appeared to be the most prevalent emotional/social difficulties affecting 33.3% of patients each. Although physical aggression, perseveration, and initiation problems were frequent, verbal aggression was the most common overt behavior exhibited by 27.8% of patients. These changes were widespread and not confined to patients with high-grade tumors or specific tumor locations, emphasizing the need for comprehensive assessment and management for all patients. A strong association between the occurrence of epileptic seizures and increased behavioral disturbances was also seen, suggesting a potential causal link. Moreover, tumors situated in the frontal lobe were specifically associated with an increased likelihood of behavioral changes. The investigation also found a moderate level of agreement between patient self-reports and clinician evaluations, suggesting that many patients with primary brain tumors (PBTs) possess adequate cognitive abilities to provide reliable self-assessments. Meanwhile, family member proxy ratings displayed a stronger agreement with clinician assessments, drawing attention to the valuable insight that family members may provide. Although a significant correlation was observed between behavioral shifts and functional status, the directionality of this relationship remains unclear [12].

A similar study done by Gregg et al. [13] documented behavioral changes in 28 patients with low-grade frontal tumors, alongside 27 patients with non-frontal tumors, utilizing the same standardized assessment tools. The results revealed notable rates of apathy (46%), disinhibition (58%), and executive dysfunction (62%). Individuals harboring tumors in the frontal region demonstrated a distinct pattern of rating themselves higher on behaviors associated with frontal systems than those with tumors in other areas. Additionally, a significantly larger number of patients with frontal tumors reported clinically significant issues with disinhibition. Family members of these patients also perceived higher levels of apathy in their loved ones compared to families of patients with tumors in non-frontal areas. However, it is important to note that a considerable portion (at least 40%) of both patients and their families reported clinically significant levels of apathy and executive dysfunction, regardless of the tumor's location. Lastly, clinically significant levels of anxiety were reported more frequently by patients with frontal tumors compared to those with tumors in other regions [13].

Anxiety and depression are consistent challenges that persist throughout the disease course of brain tumor patients. These emotional challenges are continuous yet fluctuating, with peaks often observed around critical junctures such as diagnosis, surgery, and recurrence. Research indicates that anxiety is remarkably prevalent among those awaiting neurosurgery, with over 80% experiencing some degree of anxiety and over 55% reporting severe anxiety. This highlights the importance of proactively addressing anxiety through routine screening and appropriate interventions as it can substantially affect patients' overall well-being, coping strategies, and even their experience of postoperative pain. Moreover, depression is a common concern for brain tumor patients, with reported rates varying from 5% to 44%. Alarmingly, both anxiety and depression tend to be under-recognized and under-treated in this population, indicating a crucial need for improved identification and support. This is particularly worrisome given the link between depression and poorer survival outcomes in some studies [14].

The diagnosis and treatment of brain tumors may also induce a wide range of psychosocial challenges, due to a combination of neuro-oncologic fears and distress symptoms. Patients frequently report a loss of relational closeness, decreased social activity, and a sense of altered identity. These feelings are compounded by the recognition that family and friends may be less willing to share caregiving responsibilities as the patient's behavior becomes more challenging [10]. These challenges chiefly manifest as anxiety, depression, and other forms of psychosocial distress exacerbated by various other neurological symptoms associated with brain tumors. Studies have consistently shown that brain tumor patients experience moderate-to-high levels of psychosocial burden both prior and after treatment. This burden is further aggravated by the loss of independence, difficulties in resuming work, changes in interpersonal dynamics, and the social stigma associated with having a brain tumor. Furthermore, the disruption of social roles within families and communities can lead to a vicious cycle of social withdrawal and isolation [15].

Social cognitive functioning involves the ability to perceive, interpret, and respond to the mental and emotional states of others, being crucial for effective social interactions. Research on social cognitive functioning in brain tumor patients presents varied findings. Some studies indicate that these patients may exhibit considerable social-cognitive deficits even before treatment begins, suggesting that the tumor itself contributes to these impairments. These challenges may persist or even intensify after treatment, particularly following surgery, or radiation therapy. Conversely, other studies have observed only mild or short-term social cognitive impairments, where patients might experience a temporary decline in social cognitive abilities immediately following surgery, with gradual improvement over the subsequent months. This recovery could be linked to brain plasticity, though further research is needed to explore the underlying processes. Despite the varied results, there is an emerging consensus that social cognitive impairments are clinically significant in brain tumor patients. These impairments can lead to substantial difficulties in social interactions, which may increase psychosocial stress and negatively affect QoL [15].

Types and locations of brain tumors' effect on emotional impact: a multi-study review

**Figure 2:**
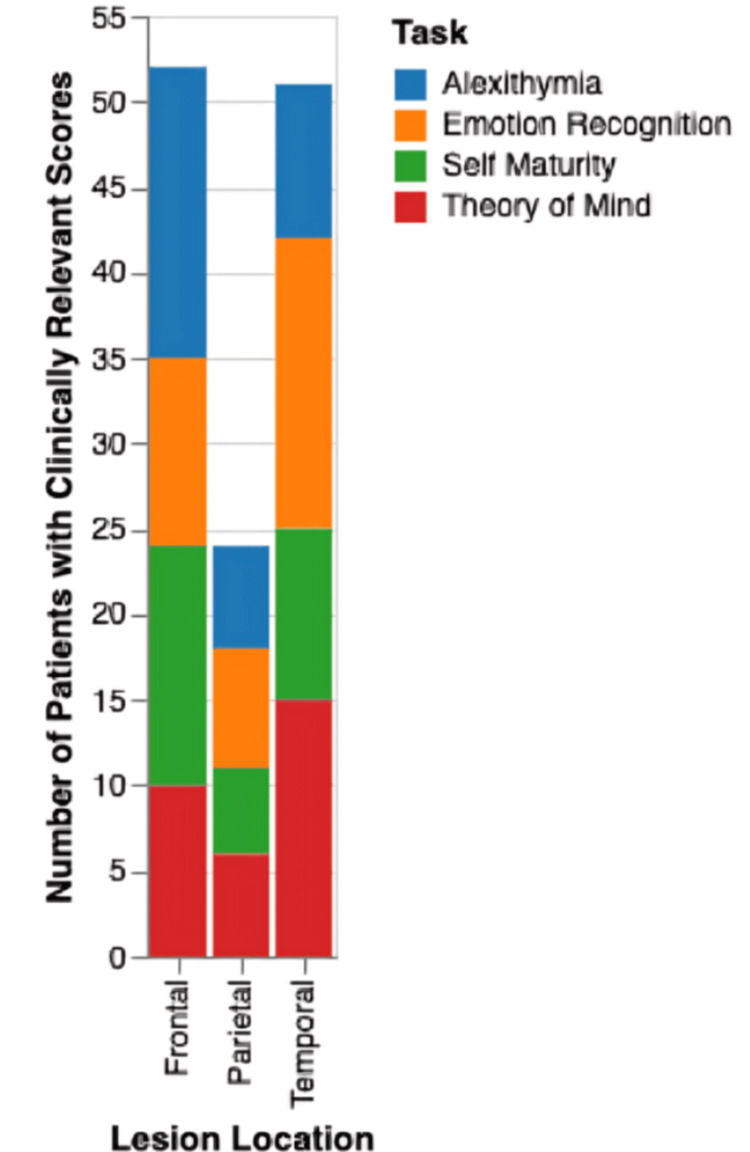
Cognitive Impairment by Lesion Location Image Credits: Qalbe Muhammad; tool used: Microsoft PowerPoint

Brain tumors can manifest in various locations within the brain, each associated with distinct emotional and cognitive changes. Studies by Campanella et al. [16,17] examined the cognitive performance of 66 patients diagnosed with HGGs, low-grade gliomas (LGGs), and meningiomas. Participants completed four tasks designed to assess various levels of emotional and intentional processing. Their performance was evaluated before surgery, immediately after surgery, and again four months later for the LGG group. The results revealed distinct patterns across the patient groups as depicted in Figure 2.

Patients with temporal lobe lesions particularly its anterior regions, the insula, and the amygdala had the highest percentage of clinically relevant scores on tasks related to emotion recognition. They would exhibit difficulties identifying and interpreting facial expressions, especially those conveying fear. This can lead to misinterpretations of social cues, inappropriate reactions, and challenges in navigating interpersonal relationships. Furthermore, tumors affecting the temporoparietal junction and superior temporal gyrus regions, typically involved in eye gaze monitoring and action processing, can impair the “Theory of Mind”, the ability to understand others's mental states and intentions. This deficit can lead to difficulties in social interactions, as individuals struggle to accurately interpret the thoughts and feelings of others. Frontal lobe tumors, on the other hand, were often associated with alexithymia, a condition characterized by difficulty identifying and describing one's own emotions. They may also experience decreased self-maturity, leading to impulsive behavior, reduced empathy, and a higher risk of personality disorders. The ventrolateral prefrontal cortex (PFC), which is crucial for executive control and goal-directed behavior, seems to play a key role in these changes [16,17].

**Figure 3:**
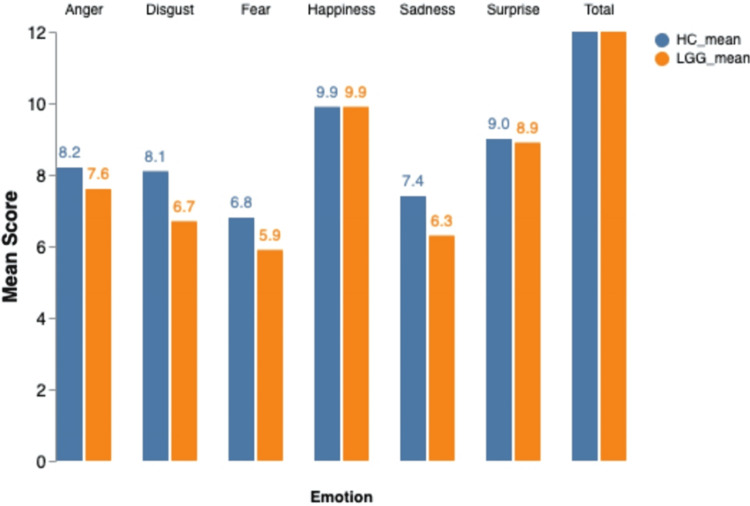
Emotion Recognition in Low-Grade Glioma Patients vs Healthy Controls LGG: Low-Grade Glioma; HC: Healthy Controls; Image Credits: Qalbe Muhammad; tool used: Microsoft PowerPoint

Case studies and limited quantitative research hint at a correlation between tumors in the frontal lobe, limbic system, and cerebellum and changes in personality related to aggression. This aggression can manifest both outwardly, directed towards others, and inwardly, potentially culminating in suicidal thoughts and even completed suicide. Notably, approximately 80% of tumor patients exhibiting psychiatric symptoms have tumors situated in the frontal lobes or limbic system, suggesting a heightened vulnerability to personality changes in individuals with these tumor types. Importantly, these personality changes often prove persistent and deviate significantly from the individual's pre-illness personality patterns [18]. The nature of the brain tumor itself plays a crucial role in shaping emotional experiences. In cases of LGG, a slow-growing brain tumor originating from glial cells, emotional functioning can be compromised. A study published in Neuro-Oncology [19] investigated emotion recognition in a group of 121 patients with LGGs and found that these patients exhibited impairments specific to negative emotions such as anger, disgust, fear, and sadness. Around half of the participants had diffuse oligodendrogliomas, while the other half had diffuse astrocytomas, with the majority of the tumors located in the frontal lobe. A comparative bar chart based on the data from this study seen in Figure 3 compares the emotion recognition scores of LGG patients and health controls (HCs). These findings clearly demonstrate the significant difference in emotion recognition between LGG patients and HCs, emphasizing the importance of recognizing and addressing the emotional burden that LGGs place on individuals. Interestingly, location, including lateralization, size, and histopathological characteristics, appeared to have a limited impact on emotion recognition in LGG patients, suggesting the potential role of brain plasticity in adapting to slow-growing tumors. Tumor infiltration into specific regions within the frontal lobe involved, such as the insula, anterior cingulate cortex, lateral PFC, and orbitofrontal-ventromedial PFC, showed insignificant differences in emotion recognition [19].

In addition to specific emotional deficits, brain tumors may also induce wider-ranging neurological and neurocognitive symptoms. Malignant gliomas, characterized by their aggressive growth, are often associated with headaches, seizures, motor deficits, and sensory changes, related to tumor location and increased intracranial pressure. These tumors can disrupt extensive brain networks, resulting in difficulties with higher-order thinking, memory, and language. This may affect planning, problem-solving, recalling information, and communicating effectively, further complicating daily life [20]. Furthermore, studies show that malignant tumors are linked to a greater degree of psychological distress, encompassing depression, anxiety, PTSD symptoms, and somatic complaints. This heightened distress can greatly affect patients’ QoL, their willingness to adhere to treatment plans, and their overall sense of well-being. One study found that up to 48% of patients with malignant brain tumors exhibit signs of depression and anxiety, with 56% reporting elevated distress during their hospital stay. Notably, patients with intracranial tumors often experience higher levels of emotional distress than those with other cancer diagnoses. This may be due to the dual burden of facing both an oncological and a neurological disease [21]. In the context of malignant gliomas, the impact of surgery itself may add another layer of complexity to emotional well-being. While surgery aims to remove as much of the tumor as possible, it inherently carries the risk of disruption of brain networks crucial for emotional processing. A study by Sinha et al. [22] investigated this phenomenon, focusing on how glioblastoma surgery affects emotion recognition. Their findings revealed that patients often experience a decline in their ability to accurately identify emotions from facial expressions following surgery. This impairment was specifically associated with reduced integrity of white matter tracts in the right hemisphere, including the inferior longitudinal fasciculus (ILF) and inferior fronto-occipital fasciculus (IFOF), which are crucial for visual and emotional processing. Thus, careful consideration of the potential cognitive and emotional consequences of surgery is needed, alongside its oncological benefits [22].

Studies have investigated the relationship between the disconnectome, which refers to the pattern of disrupted neural connections caused by a tumor, and the occurrence of depressive symptoms in patients with glioma. These investigations have revealed that specific brain regions and networks are associated with both the presence and absence of depressive symptoms. The limbic system, involved in emotional processing and memory, appears to play a central role in this phenomenon. Notably, structures within the limbic system, such as the fornix and uncinate fasciculus, have been linked to both severe depressive symptoms and the absence of such symptoms. Thus, showcasing how brain tumors may also remodel neural circuits involved in mood regulation, causing both increased and decreased activity in different brain regions. This dynamic interaction between tumor and brain highlights the complexity of emotions in brain tumor patients and the need for further research into underlying mechanisms [23].

The emotional and psychological effects of brain tumors are not limited to adults as children with brainstem tumors face their own unique challenges. Research indicates that these young patients, especially those with tumors in the pontine and midbrain areas, often display a variety of behavioral and emotional difficulties. These can include problems with activity levels, school performance, social interaction, and even increased aggression. These findings emphasize the critical need to address the emotional and psychological well-being of children with brain tumors to reduce the impact of these challenges in their development [24].

Research methodologies used in brain tumor and emotion studies

Researchers have used a variety of methodologies to investigate the link between brain tumors and emotional processing, with functional neuroimaging studies playing a central role. These studies examine neural activity in response to emotional stimuli. Structural and functional MRI (fMRI), diffusion-weighted imaging, perfusion MRI, and magnetic resonance spectroscopy (MRS) are used to assess tumor morphology, physiology, and impact on brain function. These methods help to evaluate cognitive impairment at diagnosis and post-treatment [25]. Functional neuroimaging has revealed associations between brain activation patterns, coping strategies, and psychosocial outcomes in pediatric brain tumor survivors. MRS on the other hand uses principles of magnetic resonance to measure metabolites involved in brain function. Researchers can use this non-invasive method, to gather information regarding energy use, cell activity, and neurotransmitter levels in disrupted and compensating brain areas. By measuring these changes, insights into how tumors may affect emotional well-being can be established, along with developing personalized interventions [26]. However, tractography, while useful for detecting white matter connections, faces challenges in achieving anatomical accuracy, particularly in tumor and traumatic brain injury cases. Nevertheless, these approaches have shed light on how brain tumors can disrupt emotional-cognitive processes, revealing alterations in brain regions such as the amygdala, PFC, and anterior cingulate cortex. These findings are significant in understanding how brain tumors might lead to emotional dysregulation, manifesting as mood swings, anxiety, or depression in patients [27].

A recent randomized controlled trial evaluated the effectiveness of the Tele‐MAST (Making Sense of Brain Tumor) program, delivered via videoconferencing, in improving mental health and QoL for individuals with PBTs, compared to standard care. The study involved adults with PBT who were experiencing at least mild distress, alongside their caregivers. Participants were randomly assigned to either the 10‐session Tele‐MAST program or standard care, and their mental health and QoL were assessed before the intervention, immediately after, and at six-week and six-month follow-ups. The results showed that the participants of Tele-MAST had significantly lower depressive symptoms immediately after the intervention and at the six-week follow-up, compared to those receiving standard care. They were also nearly four times more likely to experience a clinically significant reduction in depression. The participants reported better global and emotional QoL and lower anxiety levels at these same time points. By the six-month follow-up, participants continued to show improvements in mental health and QoL compared to their pre-intervention status. Although the Tele-MAST program proved to be effective, there were no significant benefits of the intervention for caregivers [28].

However, the interpretation of these studies is often complicated by several challenges. Variability in the emotional tasks used across studies can make it difficult to draw consistent conclusions as different tasks may activate different neural networks. Additionally, individual differences among participants, such as variations in emotional reactivity or baseline cognitive function, can introduce further complexity. These factors can lead to inconsistencies in findings and make it challenging to generalize results across the broader population of brain tumor patients. Another critical issue is the limitations in statistical power and sensitivity in detecting subtle emotional processing changes. Small sample sizes, often necessitated by the rarity of certain brain tumors, can reduce the ability to detect significant differences in emotional processing between patients and healthy controls. Moreover, the nature of emotional processing, which involves multiple overlapping neural circuits, requires sophisticated and carefully designed studies to isolate the specific contributions of different brain regions [29].

Despite these challenges, the existing literature showcases the significant impact of emotional processing on learning and memory, particularly in the context of cognitive deficits seen in brain tumor patients. Emotional content is known to enhance long-term memory retention, a phenomenon that is linked to the amygdala's role in modulating memory processes. In patients with brain tumors, disruptions in these emotional-cognitive networks may contribute to the cognitive impairments often observed, such as difficulties with memory, attention, and executive function. Researchers emphasize the importance of understanding each method's limitations to avoid pitfalls and misinterpretation of results [30]. Further research is needed to clarify the specific mechanisms through which brain tumors affect emotional processing and to develop targeted interventions. This includes exploring the role of different emotional states, such as fear, happiness, or sadness. By advancing our understanding of these complex interactions, the emotional and cognitive challenges individuals face with brain tumors can be understood better, ultimately improving their QoL.

Investigating the neural mechanisms underlying emotional regulation

The structural and functional integrity of specific brain regions involved in emotion processing serve as the main neurobiological components in emotional modulation. Brain tumors may interfere with the normal functioning of these regions along with their neurotransmitter systems and neural networks. These disturbances can be attributed to both direct and indirect effects of the tumor on brain structures. Direct effects include the pressure exerted by the tumor on surrounding structures, direct infiltration of brain tissue, blockage of cerebrospinal fluid, and displacement of brain tissue potentially leading to herniation. While compression and infiltration often result in signs/symptoms referable to a specific region, herniation and obstruction of CSF flow tend to cause symptoms of increased intracranial pressure (headache, nausea, altered neurocognitive functioning). Indirect effects result from altering neurotransmitter levels or treatment modalities. Understanding these mechanisms is crucial for developing effective interventions aimed at improving emotional outcomes [31-33]. 

Primary gliomas in adults are commonly located in the frontal and temporal lobes but may also involve multiple lobes. Emotional processing and regulation are primarily mediated in the amygdala, PFC, and anterior cingulate cortex [34,35]. Tumor pathology can lead to direct damage or compression of these regions, altering their structural and functional integrity. In the case of gliomas, their tendency to infiltrate neighboring tissue can negatively affect neural pathways [31]. The amygdala plays a vital role in the perception of fear, threat, and anxiety [36]. Meanwhile, the PFC, specifically the dorsolateral PFC, is integral for cognitive control over emotional responses. It does so by downregulating amygdala activity, thereby reducing negative emotional reactions [37]. Similarly, the anterior cingulate cortex is essential for emotion regulation through its connectivity with the amygdala [38]. Disruptions in these circuitries may lead to heightened emotional dysregulation, as evidenced by increased amygdala reactivity and decreased PFC activity [39]. Studies utilizing fMRI have demonstrated that effective emotion regulation is associated with functional connectivity and coordinated activity across a network of these regions. This connectivity is hindered in brain tumor patients, thus impeding the ability to engage in adaptive emotion regulation strategies, ultimately increasing emotional distress [40]. Patients may find it hard to reframe negative situations and cope healthily, leading them to rely on unhelpful coping mechanisms such as dwelling on their problems [41].

The psychological stress that comes hand-in-hand with a cancer diagnosis and its treatment can intensify the emotional impact of brain tumors. Patients often grapple with anxiety, depression, and other emotional difficulties, further complicating their overall emotional state. Research shows that brain tumors can elevate levels of distress and emotional volatility, which may be mediated by changes in neurobiological pathways involving neurotransmitters [32,33]. The interplay between neurotransmitters, such as gamma-aminobutyric acid (GABA), glutamate, serotonin, and dopamine, within neural circuits involved in emotional regulation is complex, and disruptions can lead to significant emotional disturbances. The balance between inhibitory neuronal transmissions via GABA and excitatory neuronal transmissions via glutamate is necessary for maintaining emotional stability. Increased levels of glutamate have been implicated in the pathophysiology of anxiety and mood disorders. Brain tumors can exacerbate this imbalance through their metabolic demands and the inflammatory responses they provoke [42,43]. Dopamine is noteworthy for its involvement in the brain's reward pathways and is critical for motivation and pleasure. Brain tumors can influence dopamine metabolism and receptor expression, leading to dysregulation of dopaminergic signaling, associated with various mood disorders and emotional instability [44,45]. For instance, dopamine receptor expression has been seen to be altered by glioblastoma, contributing to emotional and cognitive deficits in affected patients [46]. Serotonin, on the other hand, has been implicated in modulating emotional responses, with research indicating that alterations in serotonergic function can lead to changes in connectivity between the default mode network (DMN) and other brain regions [47]. DMN, typically engaged during moments of rest and introspection, displays unusual connectivity patterns in individuals who struggle with regulating their emotions. This suggests that neurochemical imbalances, regularly observed in brain tumor patients, strongly contribute to their common comorbidities, including anxiety and depression [48]. Individual genetic predispositions may also play a role in modulating emotional responses to brain tumors. Variations in genes associated with neurotransmitter systems, such as the vesicular monoamine transporter 1, contribute to individual differences in emotional processing. Therefore, certain patients may be more vulnerable to emotional dysregulation due to their inherent genetic makeup, which can compound the neurobiological changes caused by the tumor and its treatment [49].

Brain tumors can induce a state of chronic neuroinflammation, which has also been linked to alterations in neurotransmitter metabolism and neural circuitry associated with emotional processing. The persistent inflammatory cytokines released in response to tumor growth disrupt neurotransmitter systems. Neuroinflammation has been shown to alter the functioning of the PFC and amygdala, leading to increased anxiety and depressive symptoms [50]. Furthermore, the hypothalamic-pituitary-adrenal (HPA) axis, responsible for the body's stress-response system, can also be thrown off-balance by the stress of the tumor itself. This can cause a surge in cortisol levels, contributing to even more emotional turmoil [51].

The emotional consequences of brain tumors are not homogenous and are influenced by numerous factors, including tumor type, location, and therapeutic interventions. Patients with low-grade tumors may encounter distinct emotional challenges compared to those with high-grade tumors, reflecting the varying degrees of disruption to the neural circuits governing emotional processing [52,53]. Additionally, certain treatment methods, such as radiation therapy, can exert enduring effects on brain regions implicated in emotional processing. Research has demonstrated that radiation targeting the amygdala-orbitofrontal network can be associated with impairments in emotion recognition. Immunotherapy and radiation treatments have also been associated with neuroinflammation. Thus, it is imperative for a nuanced understanding of how tumor pathology and treatment interact to influence emotional outcomes [43,53].

The inherent ability of neuroplasticity may facilitate the development of compensatory mechanisms that can potentially mitigate some emotional deficits. Neurofeedback interventions, for instance, have demonstrated promising results in assisting individuals to regulate their emotional responses by promoting adaptive neural patterns. These interventions can foster emotional resilience and enhance overall emotional well-being, paving the way for therapeutic strategies that leverage neuroplasticity to support emotional functioning in this population [54].

The influence of psychosocial distress and coping strategies

Patients with brain tumors have complex emotional needs that are integral to their overall care. Psychosocial factors significantly shape the emotional distress and coping mechanisms of brain tumor patients, affecting how they experience and manage their illness. This necessitates a comprehensive approach to patient management that includes psychosocial interventions and supportive treatment services. The effectiveness of these interventions has been steadily expressed in the literature, with clear recognition that they play a crucial role in enhancing the QoL for both patients and their families. One of the most important influences on a patient’s emotional well-being is the presence and strength of their support system. Family, friends, and healthcare providers play a vital role in providing comfort and reassurance, to reduce feelings of fear, anxiety, and isolation. Patients who feel supported are often better able to cope with the emotional demands of their condition, finding it easier to maintain a positive outlook and manage their stress. In addition to these factors, a patient’s resilience and past experiences with adversity can influence how they cope with their diagnosis. People who have successfully dealt with previous challenges may have developed a stronger sense of resilience, helping them to find strength during difficult times, as opposed to those with less experience who may feel overwhelmed by their diagnosis [55,56].

A comprehensive study explored these psychological needs through focus groups that included 15 adult brain tumor patients (average age of 46, 53% female). Each 90-minute session was guided by a trained moderator who asked questions about the patient's experiences and needs throughout their treatment journey. The conversations were analyzed using NVivo software, identifying two key themes, emotional response to stressors and existential concerns, from which 14 specific ideas were grouped into three categories: fear, despair, and resilience. These categories represented between 31.4% and 34.7% of the discussion, highlighting the emotional journey of patients as they navigate their diagnosis. The study highlights the need for including patient perspectives when designing neuro-oncology care programs, as addressing emotional distress, and building resilience should be key components of care [57].

A novel investigation aimed to characterize these changes through subjective, observer-rated, and clinical measures. The research involved 44 patients undergoing neurosurgery for brain tumors and 26 patients who had undergone spinal surgery as a control group. The patients completed the Hospital Anxiety and Depression Scale and a Subjective Emotional Change Questionnaire, while observers who knew the patients well provided ratings using the Iowa Rating Scale of Personality Change. The findings indicated that brain tumor patients experienced significantly greater changes in their emotional responses following surgery, particularly in anger, disgust, and sadness, compared to the control group. Observers also noted notable personality changes in the tumor patients, including increased irritability, impulsivity, moodiness, inflexibility, and a heightened tendency to feel overwhelmed. Despite these changes, anxiety and depression levels did not differ significantly between the two groups. The study concluded that neurosurgical resection of a brain tumor leads to alterations in patients' emotional experiences and personality traits, though these changes are not necessarily associated with heightened anxiety or depression [7].

The effectiveness of psychosocial interventions and supportive care

­It is important to magnify the numerous social, emotional, and spiritual concerns that patients and their loved ones frequently experience, which often go unrecognized in clinical settings. This gap in care demonstrates the need for healthcare providers to proactively screen patients and families for these psychosocial needs and then collaborate with them to address these concerns and bridge that divide [58]. A study showcases that providing tailored supportive care interventions can lead to substantial HRQoL improvements for these patients. The study reveals that anxiety and depression rates are high among patients diagnosed with a brain tumor, but these can be reduced through interventions such as counseling, support groups, or psychoeducation [59]. Moreover, psychosocial care is increasingly recognized as a vital part of holistic cancer care and should ideally be a routine component in treatment paradigms [60].

Structured psychosocial interventions have been empirically proven to be effective. For example, a study suggested that using dependency care theory as the basis for a post-surgical home care intervention significantly enhanced self-care abilities and reduced caregiver burden. This aligns with the broader literature suggesting that focused psychosocial support can help improve patient and caregiver experiences, leading to better overall health outcomes. This study underscores the need for both psychosocial and physical rehabilitation interventions throughout treatment (from a long-term perspective), as fluctuations between hopefulness and despair were part of living with metastases [61]. Accessibility to psychosocial support services, in addition to structured interventions, is crucial. The study further describes barriers that prevent patients and families from utilizing such services, including a lack of awareness about available options and the failure to integrate psychosocial care into routine oncology practice. Socioeconomic status plays a major role, as those with a higher socioeconomic status often have better access to resources such as specialized medical care, mental health services, and support groups. Telehealth is expanding its reach through communication and helping people improve their mental health. Research has found that telehealth interventions can be effective, but more studies are needed to assess their long-term effectiveness. Thus, these tools may offer a valuable opportunity for brain tumor patients to access care, especially those living far from health facilities. Patients should be able to assess the effectiveness of such tools, as their usefulness depends on patients' familiarity with them [62]. These resources can help patients feel more in control of their situation and be better equipped to handle the emotional challenges of their illness. The authors advocate for a more systematic approach to clinical psychosocial care that includes training healthcare professionals to recognize and respond effectively to the distress experienced by brain tumor patients. A multicenter study supported this approach, as its data revealed significant disparities in the provision of psychosocial care across different healthcare settings [63,64].

The treatment experiences of patients with a brain tumor and their associated side effects also impact their emotional needs. Research shows that aggressive treatments lead to an increase in stress levels among patients; thus, comprehensive psychosocial care can help alleviate this distress. It is essential to consider specific psychological disorders, such as depression and anxiety, when providing interventions that address the unique emotional needs of brain tumor patients [65]. This detailed understanding of patient needs is essential for the development of successful psychosocial assistance plans.

Finally, the impact of caregivers on the psychosocial health of brain tumor patients should not be underestimated. Caregivers often experience overwhelming emotional pain, which can hinder their ability to provide support. Caregiver burden - evidenced by high levels of anxiety and depression revealed in studies like those mentioned here - indicates that the psychosocial needs of this population also require attention. Caregiver emotional well-being is closely linked to patient outcomes; therefore, interventions to alleviate caregiver distress may lead to indirect benefits for the patient's overall functioning [66]. Furthermore, the importance of communication in psychosocial care cannot be overlooked. It should be noted that patients often consider nurses pivotal in managing their signs and symptoms, yet nurses' contributions to psychosocial support remain largely unrecognized [67]. Verbal interactions between nurses and patients that address psychosocial issues enhance patient satisfaction and emotional well-being [67]. This highlights the need for ongoing education and training for healthcare professionals to develop proficiency in providing psychosocial components of cancer management.

While the characteristics of a brain tumor, coupled with challenges like anxiety and cognitive difficulties, can negatively impact a patient's social and cognitive abilities, strong social support and personal resilience can act as protective factors. These positive elements, when combined with targeted therapies that focus on improving emotional skills and problem-solving abilities, can help aid patients in navigating the social and cognitive challenges that often accompany brain tumors [15].

Implications of emotional distress on treatment, QoL, and prognosis

It is evident that the psychological well-being of brain tumor patients is significantly compromised, affecting various aspects of their lives. Addressing mental health concerns should be an integral part of comprehensive care for these individuals. As research has shown, anxiety and depression are common symptoms among brain tumor patients [68]. A direct result of extreme emotional distress can lead to poorer health-related QoL, deeming determinantal effects on this population and resulting in an overall lowered survival rate [69]. This type of mental discomfort brings forth a plethora of challenges. Firstly, emotional distress can lead to noncompliance with treatment plans, thereby reducing the effectiveness of therapy [70]. The neuropsychiatric consequences associated with primary and metastatic brain tumors, coupled with the psychological burden of diagnosis, prognosis, and treatment, create a heightened risk of developing mental health disorders in these patients [65]. Mental health disorders may manifest in various ways, including, fatigue, anxiety, memory difficulties, and persistent worry [55]. The significant levels of suffering experienced by HGG patients emphasize the significance of addressing emotional well-being in these patients [71]. Research consistently demonstrates the critical role of effectively managing emotional distress in brain tumor patients. Therefore, reducing emotional distress not only enhances treatment tolerance and prognosis but also leads to improved QoL and adherence to treatment plans [72].

The intersection between emotional functioning and cognitive impairment

As mentioned earlier, alongside the myriad of emotional challenges faced by patients, cognitive deficits contribute as risk factors to the decline in QoL as well [24,64]. Research indicates that brain tumor patients experience elevated levels of emotional distress and depression by up to 90% compared with other cancer populations [73]. This is heightened when accompanied by the cognitive impairments caused by brain tumors such as difficulties with memory, attention, and processing speed. The frustration of struggling with once-easy tasks can lead to feelings of helplessness and despair, making it harder for patients to engage in activities that bring them joy or relief [53,74-78]. Emotional functioning and cognitive impairment are interlinked as emotional distress can hamper cognitive performance, whereas cognitive difficulties can lead to frustration and even more emotional distress.

Cognitive impairment hinders patients’ ability to process emotions effectively, leading to emotional and behavioral disturbances [79]. According to a study [73], increased deterioration of cognitive function is more prevalent after the treatment of brain tumors, manifesting as communication deficits and increased fatigue [75,76]. While tumors in children are associated with irritability and attention deficit, in adults, communication difficulties lead to feelings of frustration, fear, and stress due to uttering unintended words [24,75,77]. Neuropsychological tools have demonstrated a decrease in cognitive performance when there is an increase in emotional distress [24]. Anxiety due to fear of disease progression, adverse effects of treatment, and changes in identity following diagnosis negatively affected attention spans and memory retrieval processes. Meanwhile, depression, which was specifically observed in patients with malignant tumors, leads to a decrease in motivation [73,80].

Jia et al.’s [24] study reports that the coexistence of these deficits is related to the specific location and growth pattern of brain tumors, prompting the types of cognitive challenges experienced by patients [53]. For example, tumors in the frontal lobe, responsible for complex thinking skills including planning and decision-making, are usually linked to executive dysfunction [75-77]. Similarly, tumors near the temporal lobe may lead to memory impairments due to their proximity to structures critical for memory retention [76]. Treatments such as surgery, radiation, and chemotherapy themselves have direct effects on neural tissue, intensifying these symptoms [53,75,78]. Higher short-term doses of radiation increase the risk of cognitive deficits, while long-term radiation affects the emotional status of patients [78].

Addressing gaps and proposing new directions in brain tumor and emotion research

Despite the significant research on the relationship between brain tumors and emotions, several gaps require further investigation. There is a lack of studies examining emotional changes or symptoms over the entire course of the disease. Additionally, there is insufficient exploration of the neurobiological mechanisms underlying emotional changes in brain tumor patients. Moreover, studies often have small sample sizes, restricting the validity of the findings, while also excluding the crucial perspectives of caregivers and family members.

Future research should aim to conduct longitudinal investigations, tracking the changes in emotional health symptoms across the trajectory of the disease. A deeper dive into the specific pathophysiology in which various tumor types affect cognitive abilities and emotional regulation is needed. This includes assessing the relationship between emotional states and communication abilities, along with utilizing advanced neuroimaging techniques to gain a better understanding of neural mechanisms of emotional processes. Furthermore, it is imperative to prioritize the development of appropriate screening tools for depression coupled with the assessment of cognitive functions, especially within the context of surgical interventions. Evaluating the efficacy of tailored psychotherapeutic interventions for managing emotional dysfunction and the effects of chemotherapy on patients’ emotional well-being is also essential. Lastly, research must move beyond the patient alone and investigate the effects on family members' emotional well-being, with the goal of developing effective supportive strategies.

Recommendations for healthcare professionals and researchers to better support the emotional well-being of brain tumor patients

Supporting the emotional well-being of individuals affected by brain tumors requires a comprehensive approach from healthcare professionals and researchers. Creating an environment that fosters open communication is crucial. Healthcare professionals should facilitate discussions about emotional support needs and emotional health during consultations to allow patients and their families to feel comfortable expressing their fears, anxieties, and concerns regarding diagnosis, treatment, and prognosis [64,75,81].

Healthcare professionals should be properly trained to help patients and ensure their emotional needs are met by integrating psychological support services and scheduling regular sessions addressing anxiety, depression, and other emotional and cognitive challenges [64,79]. Researchers should develop strategies to overcome language and communication barriers, provide therapy for cognitive issues, and investigate the role of rehabilitation in brain tumor patients [75]. Additionally, peer support groups where patients can share experiences, relate to others, and discuss coping strategies for common psychological diagnoses and treatments can be helpful [79].

Healthcare providers and researchers must aim to create individualized care plans that include psychological assessments to identify specific emotional needs and tailor interventions accordingly. Moreover, awareness should be raised to overcome cultural, financial, and social barriers. Promoting physical activity has significant benefits for mental health. Implementing routine screening for emotional and cognitive disturbances early in the diagnosis process can help with timely intervention [64]. Utilizing digital health tools such as mobile apps and telehealth services to deliver continuous psychological support and remotely monitor emotional well-being, especially for those unable to visit healthcare facilities, has shown positive results. This approach is particularly beneficial for pediatric brain tumor patients attending school online and connecting with their peers [64]. By adopting these recommendations, healthcare professionals and researchers can create a more supportive environment that addresses the physical aspects of brain tumors and prioritizes the emotional and psychological well-being of patients.

## Conclusions

This review draws attention to the influence of brain tumors on emotions, cognitive functions, and behavior. Tumor characteristics, such as location and growth rate, along with the treatments employed, significantly influence the emotional experiences of patients. These factors can affect neural pathways and their neurotransmitters leading to disruptions in normal emotional functioning. Neurological and neurocognitive changes, including difficulties with emotion recognition, alexithymia, and social cognitive deficits, can lead to significant challenges in navigating daily life and maintaining interpersonal relationships. The presence of mood disorders, such as depression and anxiety, further exacerbate these challenges. Healthcare professionals are urged to adopt a comprehensive approach that integrates psychosocial care into routine practice. This includes screening for emotional and cognitive disturbances, providing access to structured interventions and support services, and fostering open communication. Interventional psychotherapy, rehabilitation, and other coping strategies have been explored. Further evidence is required to achieve the desired outcomes in handling the emotional, behavioral, and cognitive well-being of these patients.
